# Identification of the Replication Origins from *Cyanothece* ATCC 51142 and Their Interactions with the DnaA Protein: From *In Silico* to *In Vitro* Studies

**DOI:** 10.3389/fmicb.2015.01370

**Published:** 2015-12-10

**Authors:** He Huang, Cheng-Cheng Song, Zhi-Liang Yang, Yan Dong, Yao-Zhong Hu, Feng Gao

**Affiliations:** ^1^Department of Biochemical Engineering, School of Chemical Engineering and Technology, Tianjin UniversityTianjin, China; ^2^Key Laboratory of Systems Bioengineering, Ministry of Education, Tianjin UniversityTianjin, China; ^3^Collaborative Innovation Center of Chemical Science and EngineeringTianjin, China; ^4^Department of Physics, Tianjin UniversityTianjin, China

**Keywords:** DnaA, DnaA (IV), DNA binding, origin of chromosomal replication (*oriC*), initiation complex, *Cyanothece* ATCC 51142

## Abstract

Based on the complete genome of *Cyanothece* ATCC 51142, the *oriC*s of both the circular and linear chromosomes in *Cyanothece* ATCC 51142 have been predicted by utilizing a web-based system Ori-Finder. Here, we provide experimental support for the results of Ori-Finder to identify the replication origins of *Cyanothece* ATCC 51142 and their interactions with the initiator protein, DnaA. The two replication origins are composed of three characteristically arranged DnaA boxes and an AT-rich stretch, and the *oriC* in the circular chromosome is followed by the *dnaN* gene. The *dnaA* gene is located downstream of the origin of the circular chromosome and it expresses a typical DnaA protein that is divided into four domains (I, II, III, IV), as with other members of the DnaA protein family. We purify DnaA (IV) and characterize the interaction of the purified protein with the replication origins, so as to offer experimental support for the prediction. The results of the electrophoretic mobility shift assay and DNase I footprint assay demonstrate that the C-terminal domain of the DnaA protein from *Cyanothece* ATCC 51142 specifically binds the *oriC*s of both the circular and linear chromosomes, and the DNase I footprint assay demonstrates that DnaA (IV) exhibits hypersensitive affinity with DnaA boxes in both *oriC*s.

## Introduction

The replication initiation of bacteria requires two basic elements: the discrete origin of replication (*oriC*) for positively acting replication factors and the initiator protein (DnaA) to which other replication proteins bind, promoting origin unwinding and the subsequent initiation of DNA polymerization (Duderstadt and Berger, 2008). The initiation of bacterial chromosome replication is mediated by the initiator protein DnaA, which recognizes and specifically binds to non-palindromic, repetitive, 9-mer consensus sequences termed “DnaA boxes”. DnaA boxes are present in most replication origins in bacterial chromosomes, as well as in the replication origins of some plasmids (Messer, 2002; Mott and Berger, 2007). The replication origins vary in terms of both the size and number of DnaA boxes across different species.

The initiator protein, DnaA, is a key protein in the initiation of chromosome replication. DnaA is highly conserved among different bacteria (Fujita et al., 1990; Kaguni, 1997). The bacterial consensus DnaA box sequence, 5′-TT^A^/_T_TNCACA-3′, is highly conserved with only one or two nt differences. As for *Synechococcus* sp. Strain PCC 7942, the consensus sequence of the DnaA box is TTTTCCACA, as it was found in seven of the eleven repeats (Liu and Tsinoremas, 1996). Six DnaA boxes of *Anabaena* sp. strain PCC 7120, were predicted by Ori-Finder; their sequence is TTTTCCACA, and the assays confirmed the predicted result (Zhou et al., 2011). DnaA was assigned to four functional domains, I, II, III, and IV based on the degree of sequence similarity (Roth and Messer, 1995; Erzberger et al., 2002). The ssDNA-binding activity of DnaA domain I is weak (Abe et al., 2007); however, the interactions between domain I and several proteins, including domain I itself, as well as DnaB helicase, and the initiation stimulator DiaA, are required in order for DnaB helicase to load onto *oriC* open complex flexible linker (Weigel et al., 1999; Felczak and Kaguni, 2004; Abe et al., 2007; Keyamura et al., 2007; Nozaki and Ogawa, 2008). Domain II is highly variable in sequence and length in different bacteria (Messer, 2002). Domain III plays a major role in adenosine triphosphate (ATP) and adenosine diphosphate (ADP) binding, as well as in ATP-dependent conformational changes of the DnaA multimer on *oriC*, in binding ssDNA of the *oriC* duplex unwinding element (DUE), and in ATP hydrolysis (Katayama, 2008; Ozaki et al., 2008). Domain IV, the DNA-binding region [denoted as DnaA (IV) herein], comprises three potential α-helices that feature a highly conserved basic loop and a long connector helix (α12) linked to a helix-turn-helix (HTH) motif (α15 and α16), which is buttressed by two additional helices (α14 and α17) (Erzberger et al., 2002). In *Escherichia coli*, the DnaA protein is also a transcriptional regulator; it is autoregulated and interferes with the activity of other genes through DnaA binding at their promoter region (Messer and Weigel, 1997). DnaA may either bind to a DnaA box downstream of the promoter region to block transcription, such as the *mioC* promoter in *E. coli* (Theisen et al., 1993; Blaesing et al., 2000), or it may activate the expression of a gene such as *fliC* (Mizushima et al., 1994). Furthermore, with the respect to sporulation in *B. subtilis*, the DnaA protein not only initiates DNA replication, but it also regulates other aspects of cell activities (Veening et al., 2009).

Cyanobacteria, also called blue–green bacteria, blue–green algae, cyanophyceae, or cyanophytes, represent a large and widespread group of photoautotrophic microorganisms (Stanier and Bazine, 1977; Whitton and Potts, 2012). Cyanobacteria are the only diazotrophs that produce molecular oxygen as a byproduct of photosynthesis; they have evolved a variety of mechanisms to accommodate the activity of an oxygen-sensitive enzyme (Berman-Frank et al., 2003). As we know, nitrogen fixation has played a crucial role in marine environments where the bioavailability of nitrogen determines the level of primary productivity (Montoya et al., 2004). *Cyanothece* sp. ATCC 51142 (hereafter referred to as *Cyanothece* 51142), a marine unicellular diazotrophic strain, features a robust diurnal cycle in which the processes of oxygenic photosynthesis and nitrogen fixation are performed and separated temporally within the same cell during the diurnal cycle (Sherman et al., 1998; Welsh et al., 2008a). The complete genome of *Cyanothece* 51142 was reported in 2008, and it was the first strain of the genus to be sequenced (Welsh et al., 2008a). In addition, *Cyanothece* 51142 was the first to report that there is a linear element in the genome of a photosynthetic bacterium (Welsh et al., 2008a). However, the origin of replication could not be determined for either the circular or linear chromosome using standard GC skew and DnaA box analysis at that time (Welsh et al., 2008a). By utilizing a web-based system called Ori-Finder^1^, we have identified the locations of *oriC*s for both the circular and linear chromosomes in *Cyanothece* 51142 (Gao and Zhang, 2008b), which may provide clarity on the replication origins in *Cyanothece* 51142, as well as other cyanobacteria (Welsh et al., 2008b). Meanwhile, only experimental work will finally answer this question.

In the present study, we have amplified the putative *oriC*s of the circular and linear chromosomes of *Cyanothece* 51142 via polymerase chain reaction (PCR). We have also cloned the *dnaA* gene of *Cyanothece* 51142. Domain IV of the *dnaA* gene has been cloned into an overexpressing vector from *E. coli* for purification. According to the prediction of Ori-Finder, both of *oriC*s contain three putative DnaA boxes. Here, we characterize the interaction of the purified *Cyanothece* 51142 DnaA protein, domain IV [DnaA (IV)] with *oriC*s via gel retardation assays and a DNase I footprinting assay to provide experimental support for the prediction by Ori-Finder.

## Materials and Methods

### Bacterial Strains, Media, and Culture Conditions

*Escherichia coli* DH5a (F^-^,Φ80d*lac*ZΔM15, *rec*A1, *end*A1, *gyr*A96, *thi*-1, *hsd*R17, [r_k_^-^, mk^+^], *sup*E44, *rel*A1, *deo*R, Δ[*lac*ZYA-*arg*F]U169) (Sambrook et al., 1989) served as a host for plasmids, while *E. coli* BL21 (DE3) acted as the host for the overproduction of recombinant proteins DnaA (IV) (**Table 1**). The *E. coli* strains were grown in Luria-Bertani (LB) medium at 37°C. The plasmids and oligonucleotides used in the present study were described in **Tables 1** and **2**, respectively. Antibiotics were used in the following concentrations: ampicillin (100 μg/mL) for *E. coli*, and kanamycin (50 μg/mL) for plasmids.

**Table 1:** Bacterial strains and plasmids used in this study.

Strain/plasmid	Genotype/relevant characteristics	Source
*Escherichia coli* DH5α	*supE*44*, hsdR*17*, recA*1*, endA*1*, gyrA*96*, thi-*1*, relA*1	Novagen
*E. coli* BL21 (DE3)	Overexpression of recombinant protein His_6_-DnaA (IV)	Novagen
pET-28a(+)	Kan^r^, expression vector, His-tag coding sequence	Novagen
pEASY-T1	Amp^r^, Kan^r^, T-vector for cloning PCR-amplified fragments	TransGen
pET28a-DnaA (IV)	Expression plasmid derived from pET-28a for the Domain IV of *Cyanothece* 51142 His_6_-DnaA (IV)	This study
pEASY-*oriC*-C	Cloning plasmid derived from pEASY-T1 of *oriC* region of circular chromosome	This study
pEASY-*oriC*-L	Cloning plasmid derived from pEASY-T1 of *oriC* region of circular chromosome	This study


**Table 2:** Oligonucleotides used in this study.

Names	Sequence(5′ to 3′)^a^
*dnaA*-F	ATGACTATTTCCCCTCAATATATTTGGAACC
*dnaA*-R	TTAATTTTGATTCCGACTAGCAATATTAATG
*dnaA*(IV)-F	CG**GGATCC**ATTTCCATTTCAGGACTATCGATGA
*dnaA*(IV)-R	CC**AAGCTT**TTAATTTTGATTCCGACTAGCAATA
*oriC*-C-F	GCTCTAACTTTCGCTTCTCTTGGAAGCCAT
*oriC*-C-R	ACGGCCCGACTGACTAAAGATAAATTGCT
*oriC*-L-F	CCGCCGTAGATATTTTAACGAAGGCCCCTC
*oriC*-L-R	TTGGTAAGCAACTTGTTGAGCCGTCAACTC
Cbox1F	GTCCTGTGGGGGTCTGTGGAAAACATTAAAATTC
Cbox1R	GAATTTTAATGTTTTCCACAGACCCCCACAGGAC
Cbox23F^b^	CCTTTAAATTTTTCCACAGTTTTTCCACAGCATTA
Cbox23R^b^	TAATGCTGTGGAAAAACTGTGGAAAAATTTAAAGG
Lbox23F^b^	ATCTTGAATGTTGATAAATTTAAGTATTTATCCTCAACATTG
Lbox23R^b^	CAATGTTGAGGATAAATACTTAAATTTATCAACATTCAAGAT
EboxF^b^	GATCCTTGTTATCCACAGGGCAGTGC
EboxR^b^	GCACTGCCCTGTGGATAACAAGGATC


*^a^Restriction sites are underlined; DnaA boxes are boxed.*

*^b^Prior to use in the EMSA assay, the oligonucleotides were annealed with their complementary oligonucleotides. The boxed bases represent the DnaA box(es).*

* Ebox represents the *E. coli* DnaA box.*

### DNA Manipulations

Plasmids and DNA fragments were purified using purification kits according to the manufacturer’s protocols (TransGen, Beijing, China). The genomic DNA of *Cyanothece* 51142 was bought from the American Type Culture Collection (ATCC; Manassas, VA, USA). The *Cyanothece* 51142 *dnaA* gene was amplified using *dnaA*-F/*dnaA*-R as the primers and the genomic DNA of *Cyanothece* 51142 as the template (**Table 2**). The *dnaA* gene of domain IV of *Cyanothece* 51142 was amplified by PCR using the *dnaA* gene as the template and *dnaA* (IV)-F/*dnaA* (IV)-R as the primers (**Table 2**; **Figure 3**). To achieve high-level expression of the DnaA (IV) protein, the *dnaA* (IV) genes were subcloned into an expression vector of the pET series (TransGen) to produce (His)_6_-tagged fusion proteins in conventional *E. coli* BL21 (DE3) cells. The coding region of the *dnaA* (IV) gene was cut out by *BamH* I and *Hind* III, and cloned in frame into the T7 promotor-driven expression vector pET-28a(+) (TransGen) using the same restriction sites. The pET-28a(+) vector contains an N-terminal His⋅Tag and an optional C-terminal His⋅Tag sequence, which can be used as an affinity ligand for purification purposes. The authenticity of the pET28a-DnaA (IV) construction was verified by sequencing both strands. Enzymes were supplied by TransGen Biotech (Beijing, China) and TaKara (Dalian, China). The oligonuucleotides used for PCR were from TransGen (Beijing, China).

To clone the chromosomal replication origin regions of *Cyanothece* 51142, the putative *oriC* region of the circular chromosome located between ORF cce_1862 and the *dnaN* gene (cce_1864) (from 1,886,587 to 1,887,114 nt) (Gao and Zhang, 2008a) was amplified by PCR with primers *oriC*-C-F and *oriC*-C-R (**Table 2**). This *ori*C fragment was inserted into the pEASY-T1 (TransGen Biotech, China; **Table 1**), resulting in the plasmid pEASY-*oriC*-C; the putative *oriC* region of the linear chromosome located between 2 ORFs, cce_5168, and cce_5169 (from 373,518 to 373,849 nt) (Gao and Zhang, 2008a) was amplified by PCR with primer *oriC*-L-F and *oriC*-L-R (**Table 2**). The fragment then also was inserted into pEASY-T1, resulting in the plasmid pEASY-*oriC*-L. The two recombinant vectors were used for subcloning or as templates for *oriC* sequencing.

### DnaA (IV) Protein Purification

The *E. coli* BL21 (DE3) cells were transformed with the pET28a-DnaA (IV) plasmid. The pI value (5.67) and molecular weight (14,069.0 Da) of DnaA (IV) were predicted with ProtParam. A 100 mL culture, in LB broth supplemented with 50 μg/mL of kanamycin, was induced by the addition of 0.5 mM isopropyl β-D-thiogalactopyranoside (IPTG) at A_600__nm_ = 0.6–0.8. Incubation then continued at 20°C for 12 h. The cells were harvested by centrifugation (10,000 *g*, 5 min, 4°C). The pellet was washed twice with phosphate buffered saline (PBS) (140 mM NaCl, 2.7 mM KCl, 10 mM Na_2_HPO_4_, 1.8 mM KH_2_PO_4_, pH 7.4) and subsequently centrifuged (10,000 *g*, 5 min, 4°C). Then, the pellet was frozen at -80°C and retained until required for the further purification steps. The bacterial pellet was thawed and suspended in His-tag binding buffer (10 mM Na_2_HPO_4_, 10 mM KH_2_PO_4_, 0.5 M NaCl, 20 mM imidazole, pH 7.8) (20 mL/g of wet biomass, ice bath). The lysozyme was added to a final concentration of 1 mg/mL and the cell suspension was incubated on ice for 30 min. The cells were lysed by sonication (ice bath) for 1 h and centrifuged at 3,000 *g* for 10 min, 4°C. The supernatant was purified using a 6× His trap column according to the standard protocol of AKTA prime plus. The sample was loaded onto a Ni-NTA (Ni^2+^-nitrilotriacetate)-agarose column, previously equilibrated with His-tag binding buffer. The His-tag DnaA (IV) protein was eluted after washing with the His-tag elution buffer (10 mM Na_2_HPO_4_, 10 mM KH_2_PO_4_, 0.5 M NaCl, 0.5 M imidazole, pH 7.8). The purified DnaA (IV) protein was analyzed by sodium dodecyl sulfate (SDS)-polyacrylamide gel electrophoresis (PAGE). Protein concentrations were determined to be about 1300 μg/mL by using the BCATM protein assay kit (PIERCE). The purified DnaA (IV) protein was checked by SDS/PAGE, and the protein purity was >98%.

### SDS-PAGE

SDS-PAGE was performed according to the method established by Laemmli (1970). The purified protein was separated by PAGE (5% stacking gel, 15% separating gel). Gels were analyzed by a Vilber Lourmat Fusion 3500 Molecular Imager and using the ImageQuant software program.

### Electrophoretic Mobility Shift Assay

Unless otherwise indicated, electrophoretic mobility shift assay (EMSA) was carried out, as described by Schaper and Messer (1995). Here, we used PCR fragments encompassing the *oriC* regions, as well as the double-stranded oligonucleotides containing various numbers and combinations of DnaA boxes from the origins. For the EMSA analysis, the predicted *oriC* regions of the circular and linear chromosomes were PCR-amplified using a pair of primers: *oriC*-C-F/R and *oriC*-L-F/R, respectively (**Table 2**). In each binding reaction, PCR fragments or nucleotides were incubated with various amounts of purified His_6_-DnaA (IV) (2–20 μg) in the presence of a nonspecific competitor (poly [dI/dC]) at 37°C for 30 min in a binding buffer (20 mM Hepes/KOH [pH 8.0], 5 mM magnesium acetate, 1 mM Na_2_EDTA, 4 mM dithiothreitol, 0.2% [v/v] Triton X-100, 3 mM ATP, and 5 mg/mL of bovine serum albumin [BSA]). The bound complexes were separated by electrophoresis in 8% polyacrylamide gels (0.25× TBE, at 4 V/cm, 4°C). Then, the gels were stained by 1× SYBR Green I solution. Gels were washed and analyzed via a Vilber Lourmat Fusion 3500 Molecular Imager and using the ImageQuant software program.

### DNase I Footprinting Assay with FAM-labeled Primers

For the preparation of fluorescent FAM-labeled probes, the *oriC*s of circular and linear chromosomes were PCR amplified with Dpx DNA polymerase (TOLO Biotech, Shanghai, China) from the plasmids, pEASY-*oriC*-C and pEASY-*oriC*-L, using M13F (fluorescent 6-carboxyfluorescein [FAM]-labeled) and M13R primers. The FAM-labeled probes were purified by the Wizard^®^ SV Gel and PCR Clean-Up System (Promega Corporation, Madison, WI, USA) and they were quantified with NanoDrop 2000C (Thermo Fisher Scientific, Waltham, MA, USA).

DNase I footprinting assays were performed similar to Wang et al. (2012). For each assay, 400 ng probes were incubated with different amounts of the His_6_-DnaA (IV) protein in a total volume of 40 μL. Following incubation for 30 min at 25°C, a 10 μL solution containing about 0.015 units of DNase I (Promega Corporation) and 100 nmol of freshly prepared CaCl_2_ was added and further incubated for 1 min at 25°C. The reaction was stopped by adding 140 μL of DNase I stop solution (200 mM unbuffered sodium acetate, 30 mM of EDTA, and 0.15% SDS). Samples were first extracted with phenol/chloroform; they were then precipitated with ethanol and the pellets were dissolved in 30 μL of MiniQ water. The preparation of the DNA ladder, electrophoresis, and data analysis were the same as the methods described previously (Wang et al., 2012), with the exception that the GeneScan-LIZ500 size standard (Applied Biosystems; Thermo Fisher Scientific) was used. The protected sites correspond to the locations of the DnaA (IV)-*oriC*s complexes visualized using the Peak Scanner software v1.0 (Applied Biosystems; Thermo Fisher Scientific).

## Results

### Prediction of Cyanothece 51142 Replication Origins and Comparison of the oriC Regions in Different Cyanobacteria

**FIGURE 1:**
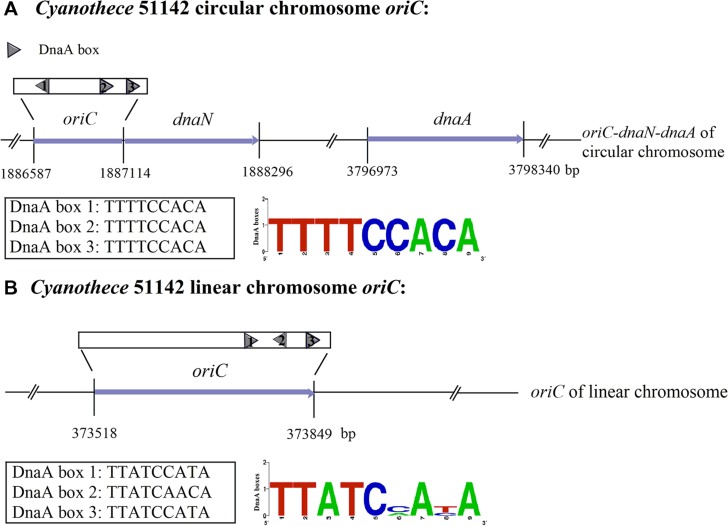
**The sequences and structure of the *Cyanothece* 51142 *oriC* regions (not to scale).** The predicted DnaA boxes 1–3 of the circular and linear chromosomes and their orientation are represented by gray pentagons. The DnaA box sequence for the circular chromosome is all TTTTCCACA, as shown in the DnaA boxes logo. The sequences of the two DnaA boxes from the linear chromosome are TTATCCATA and the other one is TTATCAACA. **(A)** The *oriC* of the circular chromosome of *Cyanothece* 51142 was predicted to be next to the start of the *dnaN* gene, and the *dnaA* gene is located downstream of the origin of the circular chromosome. The sequence of DnaA boxes is TTTTCCACA. **(B)** The *oriC* of the linear chromosome has been found in the region from 373518 to 373849 nt. The three DnaA boxes of both circular and linear chromosomes have been tagged.

Based on the *Z*-curve method, employing the means of comparative genomics, a web-based system Ori-Finder has been developed to identify *oriC*s in bacterial and archaeal genomes with high accuracy and reliability (Gao and Zhang, 2008b; Luo et al., 2014). By utilizing Ori-Finder, the locations of *oriC*s for both the circular and linear chromosomes in *Cyanothece* 51142 have been identified (Gao and Zhang, 2008a; **Figure 1**). For the circular chromosome, the *oriC* is predicted to be within the intergenic region ranging from 1,886,587 to 1,887,114 nt between the ORF cce_1862 and the *dnaN* gene (cce_1864). For the linear chromosome, the *oriC* is predicted to be within the intergenic region, ranging from 373,518 to 373,849 nt, between the ORFs cce_5168 and cce_5169. Both of *oriC*s contain three DnaA boxes, which differ by one position from the most stringent consensus sequence of the *E. coli* DnaA box TTATCCACA. With respect to the *oriC* of the circular chromosome, the DnaA boxes match perfectly to TTTTCCACA, the “species-specific” DnaA box motif for cyanobacteria, and these DnaA boxes have been found next to the start of the *dnaN* gene (**Figure 1**). A comparison of DnaA boxes from the identified *Cyanothece* ATCC51142 *oriC* regions allows us to propose the consensus (**Figure 1**). The analysis of replication origins for bacteria in DoriC^2^, a database of *oriC* regions in bacterial and archaeal genomes (Gao and Zhang, 2007; Gao et al., 2013), has also shown the conserved features associated with the *oriC* regions in the phylum cyanobacteria, such as the adjacent gene, *dnaN*, and the consensus sequence TTTTCCACA of the DnaA boxes (Gao, 2014). However, the results obtained by Ori-Finder were not sufficient to unequivocally determine the origins of replication. As such, we also performed experimental validation to confirm the results predicted by Ori-Finder.

**FIGURE 2:**
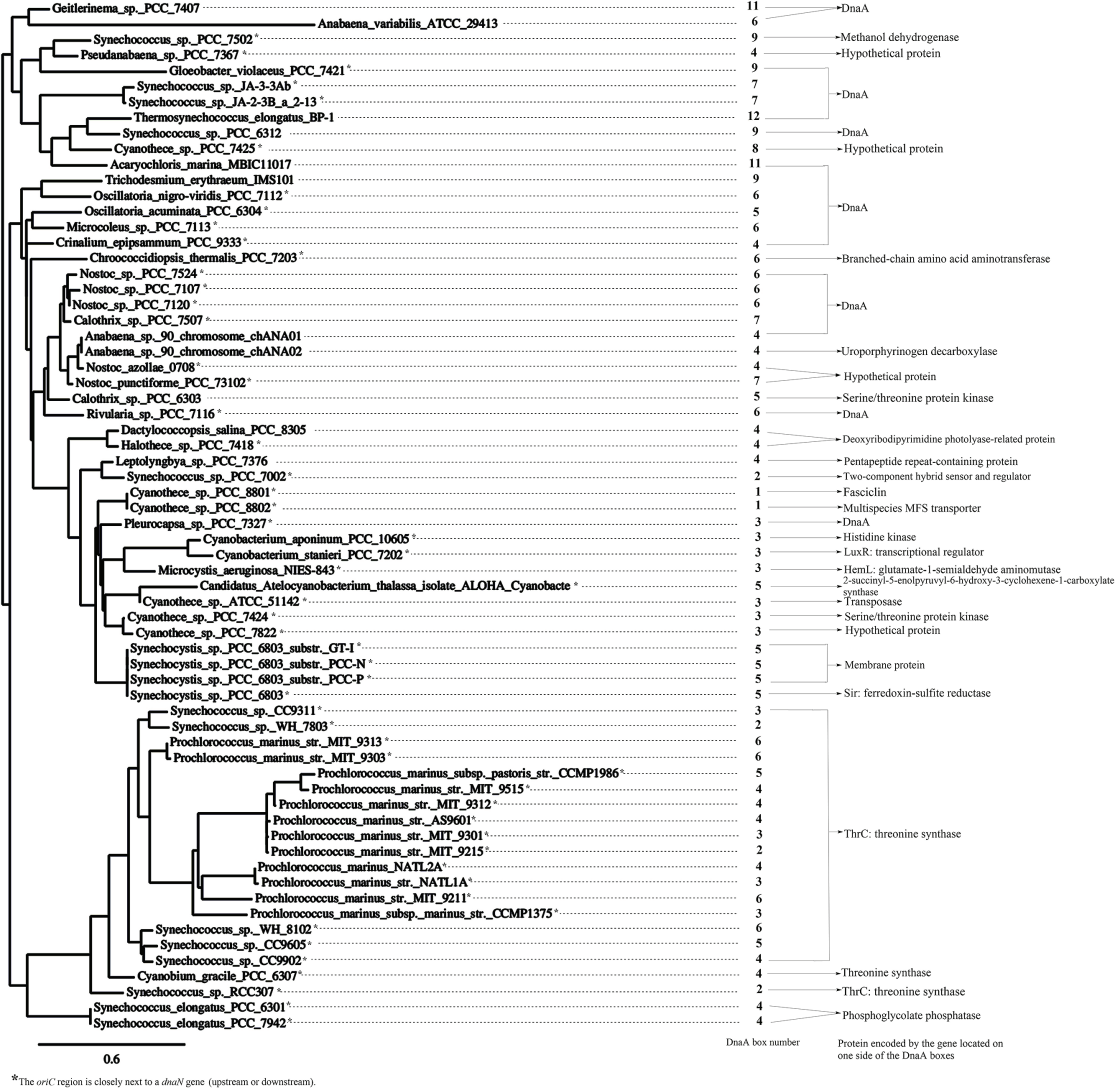
**The supplemental phylogenetic tree of cyanobacteria and information about the predicted *oriC* regions.** The figure was drawn in a similar style to that of Zhou et al. (2011), in which 38 species of cyanobacteria were shown. The phylogenetic tree was constructed with *dnaN* genes from 68 species of cyanobacteria. The sequences of DnaA boxes in most of the cyanobacteria are TTTTCCACA. Fifty-five species (indicated by an asterisk) have *dnaN* on one side, and the genes located on the other side of *oriC* are indicated on the right. Note that the *oriC* regions of the *Synechocystis* sp. PCC 6803 substr. GT-I, substr. PCC-N, and substr. PCC-P overlap the membrane protein coding sequences.

The identified *oriC* region of *Anabaena* PCC 7120 was also predicted by Ori-Finder (Zhou et al., 2011). Previously, Zhou et al. (2011) compared the *oriC* regions predicted by the Ori-Finder in 32 species of cyanobacteria. We added the other 36 species of cyanobacteria which were also predicted by Ori-Finder, and listed them in DoriC 5.0 database. Similarly, most of the 68 species of cyanobacteria have a *dnaN*-coding region nearby (**Figure 2**). Therefore, we also constructed a phylogenetic tree featuring *dnaN* gene sequences with phylogeny.fr^3^ to compare the changes of the *oriC* regions during cyanobacterial evolution (**Figure 2**). The putative *oriC* region of *Thermosynechococcus elongatus* BP-1 is bordered by genes that encode proteins of unknown functions on both sides. The range of the number of DnaA boxes at the *oriC* regions have changed, ranging from one in *Cyanothece* sp. PCC 8801 to 12 in *T. elongatus* BP-1. Furthermore, the sequences of DnaA boxes in most of the cyanobacteria are TTTTCCACA, which is the same as *Cyanothece* 51142, *Anabaena* sp. PCC 7120 (Zhou et al., 2011), and *Synechococcus elongatus* PCC 7942 (Liu and Tsinoremas, 1996). However, the *oriC* regions of *Synechocystis* sp. PCC 6803 substr. GT-I, substr. PCC-N, and substr. PCC-P overlap with the membrane protein-coding sequences; as such, it may be interesting to verify these predicted results in further experiments.

**FIGURE 3:**
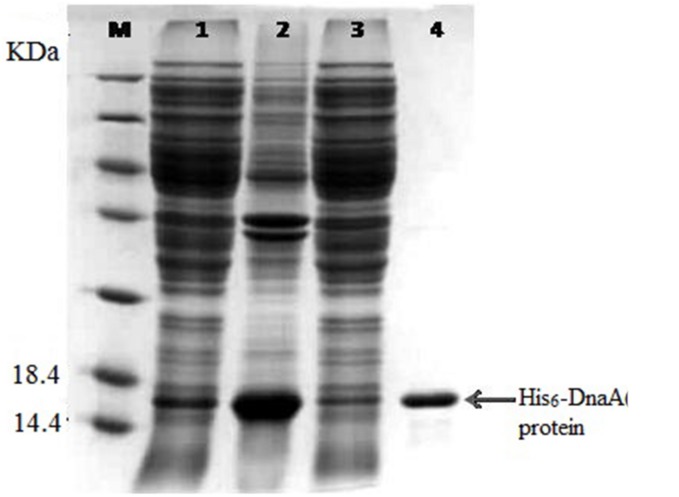
**Expression of the His_6_-DnaA (IV) in *Escherichia coli* cells.** Sodium dodecyl sulfate-polyacrylamide gel electrophoresis (SDS-PAGE) analysis of the total *Cyanothece* 51142 cell proteins. Molecular mass markers (M) are indicated on the left and the positions of the protein are indicated on the right. Lane 1, supernatant of cell lysate; Lane 2, precipitant of cell lysate; Lane 3, breakthrough peak in the affinity chromatogram; Lane 4, targeted protein in the affinity chromatogram.

### Characterization of *Cyanothece* 51142 Replication Origins, the *dnaA* Gene, and Its Product, the Binding Domain of Initiation Protein-DnaA (IV)

The predicted replication origin (*oriC*) region of the circular chromosome from *Cyanothece* 51142 is located upstream of the replication initiator gene (*dnaA*). It is composed of three putative DnaA boxes, each with a perfect match to TTTTCCACA, the “species-specific” DnaA box motif for cyanobacteria (**Figure 1**). For the linear chromosome, *oriC* is located within the intergenic region between two hypothetical open reading frames (ORFs). This identified *oriC* region is located around the minimum point of the GC disparity curve and contains three predicted DnaA boxes, each of which has no more than one mismatch from the DnaA box motif for *E. coli*: TTATCCACA. It is also observed that the *oriC* for the linear chromosome contains a reverse repeat.

The *dnaA* gene is located downstream of the *oriC* region of the circular chromosome, and it encodes a DnaA protein of 455 amino acid residues (∼52.4 kDa). Based on the structural and functional analysis of DnaA homologs, four domains of DnaA were deduced and the *dnaA* IV was found to express the DnaA IV protein of 125 amino acid residues (∼14.1 kDa). Previous studies have shown that the larger part of domain IV of the DnaA protein (the C-terminal 94 amino acid residues) was necessary and sufficient for DNA binding. To determine whether the C-terminus of the *Cyanothece* 51142 DnaA protein is responsible for DNA binding, its interaction with *oriC*s was analyzed by gel retardation assay. The PCR-amplified DNA fragment of the *dnaA* (IV) gene fused to the His⋅Tag sequence (see Materials and Methods for details) was overexpressed in *E. coli* BL21 (DE3). The fusion protein, His_6_-DnaA (IV) (∼14.1 kDa) was purified by affinity chromatography on the Ni-NTA-agarose column as described in the Section “Materials and Methods”.

### Identification of the Replication Origins from *Cyanothece* 51142

Electrophoretic mobility shift assay was performed to determine whether the His_6_-DnaA (IV) protein interacted with the DnaA boxes of the *oriC*s of *Cyanothece* 51142. The C-terminus of DnaA domain IV – namely DnaA (IV) – was used for all binding experiments given that domain IV of DnaA has been shown to be essential and sufficient for binding.

**FIGURE 4:**
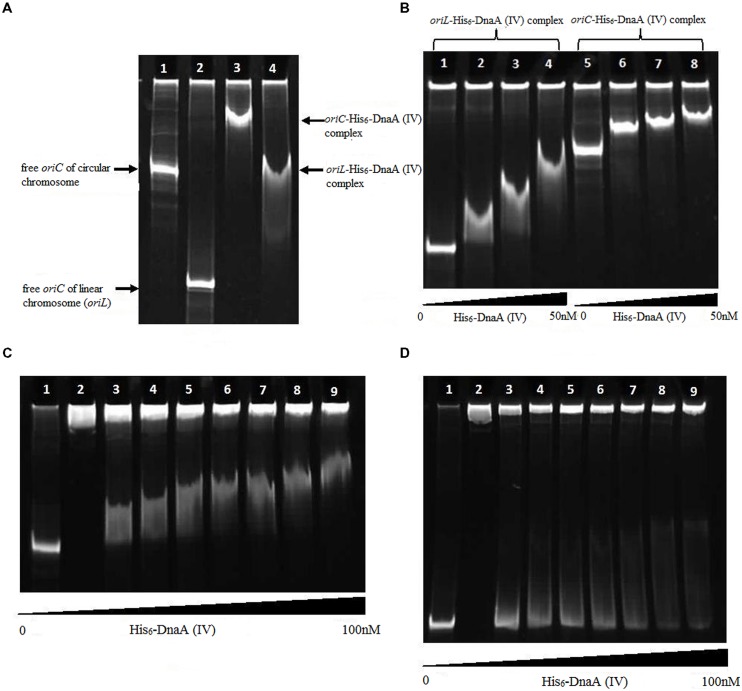
**Interaction of the DnaA (IV) protein with the *Cyanothece* 51142 *oriC.*** **(A)** Interaction of the DnaA (IV) protein with the *Cyanothece* 51142 *oriC* (5% gel). (1) Free *oriC* fragment of the circular chromosome. (2) Free *oriC* fragment of of the linear chromosome. (3) His_6_-DnaA (IV) binding to the circular chromosome *oriC.* (4) His_6_-DnaA (IV) binding to the linear chromosome *oriC.* **(B)** Interaction of the DnaA (IV) protein with the *Cyanothece* 51142 *oriC* (5% gel). (1) Free *oriC* fragment of the linear chromosome. (2–4) His_6_-DnaA (IV) binding to the linear chromosome *oriC* (10, 25, 50 nM). (5) Free *oriC* fragment of the circular chromosome. (6–8) His_6_-DnaA (IV) binding to the circular chromosome *oriC* (10, 25, 50 nM). **(C)** Interaction of the His_6_-DnaA (IV) protein with the *oriC* of the circular chromosome (5% gel). (1) *oriC* fragment of the circular chromosome [without DnaA (IV)]. (2) His_6_-DnaA (IV) protein (without the *oriC* fragment). (3–9) His_6_-DnaA (IV) binding to the circular chromosome *oriC* (3: 2.5; 4: 5.0; 5: 10.0; 6: 25.0; 7: 50.0; 8: 75.0; 9: 100.0 nM). **(D)** Interaction of the His_6_-DnaA (IV) protein with the *oriC* of linear chromosome (5% gel). (1) *oriC* fragment of the linear chromosome [without His_6_-DnaA (IV)]. (2) His_6_-DnaA (IV) protein (without the *oriC* fragment). (3–9) His_6_-DnaA (IV) binding to the linear chromosome *oriC* (3: 2.5; 4: 5.0; 5: 10.0; 6: 25.0; 7: 50.0; 8: 75.0; 9: 100.0 nM).

**FIGURE 5:**
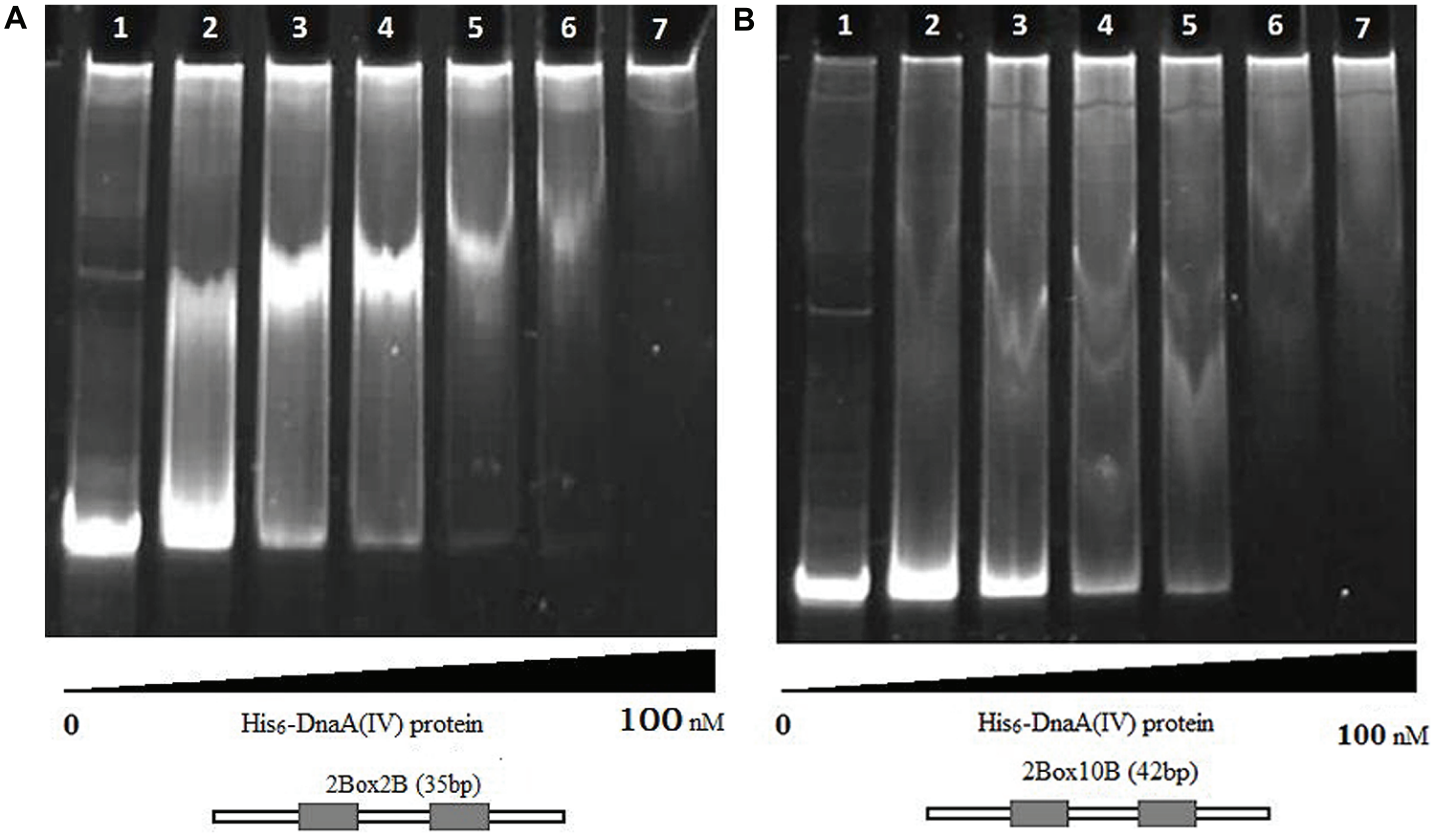
**Interaction of the His_6_-DnaA (IV) protein with the circular and linear chromosomes DnaA boxes (8% gel).** **(A)** Interaction of the His_6_-DnaA (IV) protein with the circular chromosome DnaA boxes. (1) Double-stranded oligonucleotides [Cbox23F:Cbox23R, without DnaA (IV)]. (2–7) His_6_-DnaA (IV) binding to double-stranded oligonucleotides (2: 5.0; 3: 10.0; 4: 25.0; 5: 50.0; 6: 75.0; 7: 100.0 nM). 2Box2B (35 nt): the 35-nt double-stranded oligonucleotides containing two DnaA boxes with 2 nt space. **(B)** Interaction of the His_6_-DnaA (IV) protein with the linear chromosome DnaA boxes. (1) double-stranded oligonucleotides [Lbox23F:Lbox23R, without DnaA (IV)]. (2–7) His_6_-DnaA (IV) binding to double-stranded oligonucleotides (2: 5.0; 3: 10.0; 4: 25.0; 5: 50.0; 6: 75.0; 7: 100.0 nM). 2Box10B (42 nt): the 42-nt double-stranded oligonucleotides containing two DnaA boxes with 10 nt space.

**FIGURE 6:**
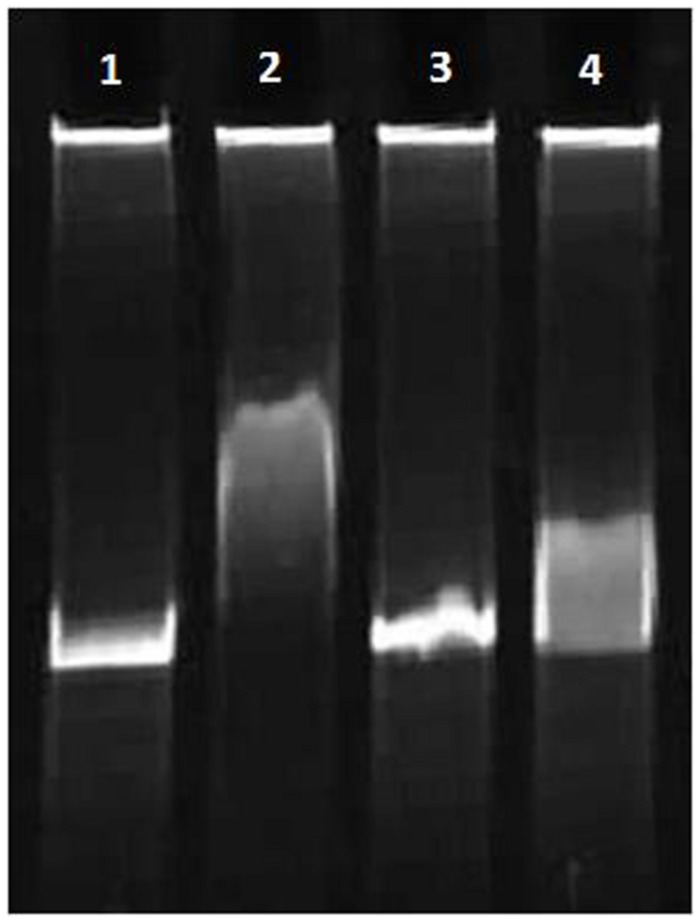
**Interaction of the His_6_-DnaA (IV) protein with linear chromosome DnaA box and *E. coli* DnaA box (8% gel).** (1,3) double-stranded oligonucleotides (1: Cbox1F:Cbox1R; 3: EboxF:EboxR). (2,4) 50.0 nM His_6_-DnaA (IV) binding to double-stranded oligonucleotides (2: Cbox1F:Cbox1R; 4: EboxF:EboxR).

**FIGURE 7:**
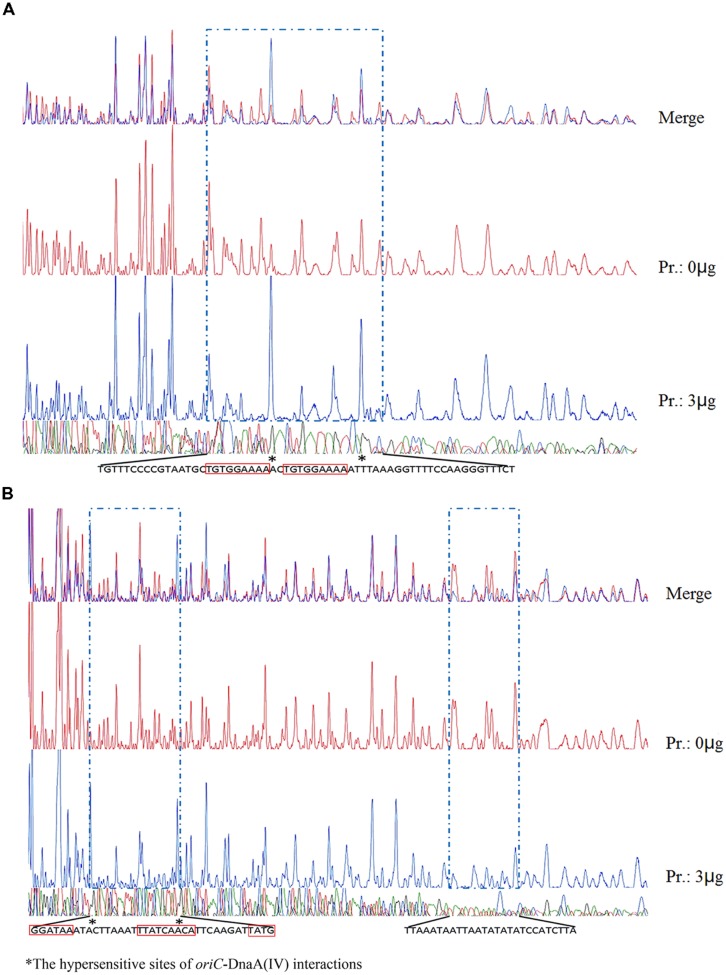
**DNase I footprint of protein His_6_-DnaA (IV) to the *oriC*s of the circular and linear chromosomes.** **(A)** Along with the increase of His_6_-DnaA (IV), a clearly protected region of 39 nt relative to the second and third DnaA boxes sites in the *oriC* region of the circular chromosome was found, although the first DnaA box was not bound to the protein [the sequences in **(A)** are reverse complementary]. The asterisks mark the hypersensitive sites, which are consistent with the locations of two DnaA boxes of the circular chromosome. **(B)** Within the protected regions of the linear chromosome *oriC*, the results from the DNase I footprinting assay revealed that His_6_-DnaA (IV) protected two specific regions- an AT-rich region, as well as a region containing two DnaA boxes and an incomplete DnaA box (TATG) [the sequences in **(B)** are reverse complementary]. The asterisks mark the hypersensitive sites, which are consistent with the locations of two DnaA boxes of the linear chromosome.

The protein–DNA complexes were analyzed by 5 or 8% native PAGE. When the PCR fragments containing each *oriC* (**Figure 4**), or oligonucleotides containing the DnaA boxes, were used to bind DnaA (IV), protein–DNA complexes were formed. As shown by the EMSA, increasing nucleoprotein complexes were observed as the DnaA (IV) concentration increased. When nucleotides with two DnaA boxes were used for binding, protein-DNA complexes were also observed (**Figures 5** and **6**). DnaA (IV) showed better affinity for the circular DnaA box motif (with two perfect DnaA boxes separated by 2 nt) than the linear DnaA box motif (with two imperfect DnaA boxes spaced by 10 nt) (**Figure 5**). Subsequently, to identify the exact DNA sequences that His_6_-DnaA (IV) protected in the *oriC* regions of the circular and linear chromosomes, a DNase I footprinting assay, combined with FAM-labeled primers using purified His_6_-DnaA (IV), was performed. Two DNA fragments (representing the entire *Cyanothece* 51142 *oriC* region of the circular and linear chromosomes) with FAM-labeled probes at the 5-end (upper strands) were incubated with different amounts of the His_6_-DnaA (IV) protein. The precipitated DNA sequences, which were protected by His_6_-DnaA (IV), were sequenced (**Figure 7**). According to the merged figure [with and without DnaA (IV)], a clearly protected region (39 nt) relative to the second and third DnaA boxes sites was found in the *oriC* region of the circular chromosome (**Figure 7A**), although the first DnaA box was not bound to DnaA(IV). Within the protected regions of the linear chromosome *oriC*, the results from the DNase I footprinting assay revealed that His_6_-DnaA (IV) protected two specific regions: an AT-rich region, as well as the region containing two DnaA boxes and an incomplete DnaA box (TATG) (**Figure 7B**). Moreover, the hypersensitive sites, which are consistent with the locations of the DnaA boxes of the circular and linear chromosomes, corroborated the results obtained with Ori-Finder and EMSA (**Figure 7**).

## Discussion

The origins of replication in *Cyanothece* 51142 have been difficult to determine using classic algorithms due to a lack of distinct patterns in strand asymmetry, although the complete genome sequence has already been determined. By utilizing the web-based system Ori-Finder, the locations of *oriC*s for both the circular and linear chromosomes in *Cyanothece* 51142 have been identified. Subsequently, we confirmed the predicted results *in vitro*. As demonstrated by EMSA and the DNase I footprinting assay, His_6_-DnaA (IV) does not clearly bind a single DnaA box; rather, it binds two DnaA boxes (the second and third DnaA boxes) from the *oriC*s of the circular chromosome, as well as three DnaA boxes (the first DnaA box is incomplete). Our results suggest that interactions of the *Cyanothece* 51142 DnaA (IV) with several DnaA boxes exhibit cooperativity.

In most bacteria, DnaA is essential for initiating chromosomal replication and recognizing the DnaA boxes near *oriC* (Skarstad and Boye, 1994). However, most of the cyanobacteria have an exceptionally low strand bias, which suggests that the process of DNA replication in these species is somehow different from that of other bacteria (Worning et al., 2006). Indeed, *Synechocystis* sp. PCC 6803 and *Prochlorococcus* lack a DnaA-binding box near *dnaA*, and they display an unusual gene arrangement in this region (Richter and Messer, 1995; Partensky et al., 1999). In addition, the *dnaA* gene of *Synechocystis* sp. PCC 6803 could be deleted without phenotypic effect (Richter et al., 1998). Furthermore, the *dnaA* genes of *Nostoc azollae* 0708, *Cyanobacterium aponinum* PCC 10605 and *C. stanieri* PCC 7202 were not detected from the genomes (data from National Center for Biotechnology Information). However, DnaA has been conserved during evolution, and its transcription is not autoregulated in the same manner as *E. coli* but light dependent instead, and follows the circadian rhythm of DNA synthesis (Richter et al., 1998). Studying the mechanism and regulation of DNA replication should reveal clues about the evolution of the DNA replication mechanism, and it will allow us to better understand the relationship between the various timing circuits involved in the circadian clock and cell division cycles.

The freshwater cyanobacteria *S. elongatus* PCC 7942 and *Synechocystis* sp. PCC 6803, have been used as model organisms for phototrophs because their transformation efficiency and growth rate are superior to those of marine cyanobacteria and their complete genome sequences have been published. In cyanobacteria, the cell division cycle is strongly light dependent, and light is the most important factor that affects the circadian clock. Most publications pertaining to the replication origins of cyanobacteria also focus on light-dependent DNA replication processes (Liu and Tsinoremas, 1996; Richter et al., 1998; Ohbayashi et al., 2013). It has been reported that for *S. elongatus* PCC 7942 and *Synechocystis* sp. PCC 6803, DNA replication depends on photosynthetic electron transport (Hihara et al., 2003; Ohbayashi et al., 2013). We successfully identified the *oriC* regions of *Cyanothece* 51142; however, it is regrettable that we do not have live cells to further assess whether or not the DNA replication of *Cyanothece* 51142 is light dependent. Knowledge about the interactions between the *Cyanothece* 51142 DnaA protein and *oriC*s may provide fresh insights into the function of this protein, as well as into the regulation of the initiation of cyanobacterial chromosome replication. Further studies are required to understand the exact mechanism underlying the phenotypes of *oriC*s of both circular and linear chromosomes. The exact mechanism underlying DnaA protein regulation in the replication, and other functions, of linear chromosome also need to be investigated.

## Conflict of Interest Statement

The authors declare that the research was conducted in the absence of any commercial or financial relationships that could be construed as a potential conflict of interest.

## Footnotes

1 http://tubic.tju.edu.cn/Ori-Finder/

2 http://tubic.tju.edu.cn/doric/

3 http://www.phylogeny.fr/simple_phylogeny.cgi
